# The Relationship Between Interoception, Alexithymia, Autistic Traits and Eating Pathology in Autistic Adults

**DOI:** 10.1007/s10803-024-06708-5

**Published:** 2025-02-14

**Authors:** Heather Westwood, Will Mandy, Rebecca Brewer

**Affiliations:** 1 NHS Lothian, Edinburgh, UK; 2 Royal Holloway, University of London, Egham Hill, Egham, TW20 UK; 3 University College, London, UK

**Keywords:** Autism, Eating disorder, Body image, Alexithymia, Interoception

## Abstract

This study investigated the mechanisms underlying disordered eating of autistic adults, by investigating associations between eating disorder (ED) symptoms and autistic traits; autism-specific atypical eating; alexithymia; interoception; and body image in autistic and non-autistic participants. Autistic adults (*n* = 196) and non-autistic adults (*n* = 206) completed online self-report measures assessing ED pathology, atypical eating, body image dissatisfaction, interoception, alexithymia, and autistic traits. Structural Equation Modelling tested a theoretical model of the relationships between these variables, and whether these relationships were moderated by autism diagnosis. Autistic adults self-reported higher ED pathology than non-autistic adults. The proposed model and the strength of the relationship between body image and ED pathology did not differ between the autistic and non-autistic groups. Atypical eating (not related to over-evaluation of weight/shape), however, was only associated with ED pathology in the autistic group. Both traditional and additional factors, including eating disturbances not driven by weight/shape concern, appear to contribute to ED pathology in autism.

**Supplementary Information:**

The online version contains supplementary material available at 10.1007/s10803-024-06708-5.

## Introduction

There is growing clinical and research interest in the relationship between autism and eating disorders (EDs; Westwood & Tchanturia, 2017). EDs are characterised by eating disturbance that results in the altered consumption or absorption of food (APA, 2013). Traditional EDs, including anorexia nervosa (AN) and bulimia nervosa (BN) include over-evaluation of weight, shape or their control as core psychopathology (Fairburn et al., 2003). In contrast, autism is a neurodevelopmental condition, characterised by social interaction and communication difficulties, repetitive, stereotyped interests and behaviours, and atypical sensory processing (APA, 2013).

While EDs have traditionally been considered as distinct categories, there is frequent diagnostic migration between disorders (Eddy et al., 2008), meaning they are better viewed as a spectrum of internalizing pathology (Forbush et al., 2017). A transdiagnostic approach which recognises that different EDs are maintained by shared psychological features (Fairburn et al., 2003), has gained empirical support (Hoiles et al., 2012; Lampard et al., 2013). EDs are also often co-morbid with other mental health conditions, including mood and anxiety disorders (Blinder et al., 2006). Comorbidities have been linked to instability of ED diagnosis (Castellini et al., 2011) and more complex and severe presentations (e.g., Hughes et al., 2013). Recently, co-occurrence of EDs and autism has received increased attention.

Several studies (Bentz et al., 2017; Pooni et al., 2012; Rhind et al., 2014; Vagni et al., 2016; Westwood, Mandy, Simic et al., 2017; Westwood et al., 2017) have assessed autism in AN using diagnostic or screening tools including the Autism Diagnostic Observation Schedule (ADOS-2; Lord et al., 2012), considered the “gold-standard” in autism diagnosis. This work evidences that autistic traits, e.g. reduced non-verbal communication, are overrepresented in EDs, particularly in AN (Westwood & Tchanturia, 2017). Cross-sectional studies report 4-53% of participants with AN meeting diagnostic criteria for autism. A systematic review and meta-analysis also found that self-reported autistic traits are over-represented in those with AN (Westwood et al., 2016).

Given that features of autism are present during early infancy (DSM-5; APA, 2013) and EDs typically develop during adolescence or young adulthood (Volpe et al., 2016), elevated rates of autism in AN point to autism increasing the likelihood of developing an ED (although see Harris et al., 2023). However, some evidence suggests ED symptoms may be qualitatively distinct in individuals with autism. In recent qualitative research, autistic individuals with AN reported that autism reinforced their ED, with the rigidity associated with autism exacerbating fixed routines and rituals around eating, which, once developed, were difficult to change (Brede et al., 2020; Kinnaird, Norton, Stewart et al., 2019). Participants also described how traditional features of AN (i.e., a desire to lose weight and body image concerns) were less relevant in the development of their ED compared to other, non-traditional motivations. Despite this, no difference in self-reported ED symptomatology has been observed between individuals with AN scoring above and below the clinical cut-off on the ADOS-2 (Westwood, Mandy, Simic et al., 2017; Westwood et al., 2017). This raises the possibility that there could be qualitatively different routes to high Eating Disorder Examination Questionnaire (EDE-Q) scores in autistic and non-autistic individuals (Brede et al., 2020). For example, for autistic individuals, ED behaviour may be associated with autistic traits, or other forms of eating disturbance, rather than over-evaluation of weight/shape. Indeed, recent work suggests that atypical eating (measured by the Swedish Eating Assessment for Autism Spectrum Disorders) is associated with both autistic traits and ED symptomatology (Nisticò et al., 2024).

While it is known that autism is over-represented in EDs, evidence for the opposite phenomenon (i.e., elevated rates of EDs in autistic populations) is sparse (Westwood & Tchanturia, 2017). However, disordered eating not characterised by over-evaluation of weight/shape is common in autism (Cermak et al., 2010; Goldschmidt & Song, 2015; Mari-Bauset et al., 2014; Råstam, 2008). There is no agreed lexicon to describe these types of eating disturbance (Goldschmidt, 2018), but issues such as food selectivity, including food refusal and a restricted repertoire of foods, are widely reported in autism (Mari-Bauset et al., 2014). Any disturbance in eating not related to over-evaluation of weight/shape is henceforth referred to as ‘atypical eating’, while eating disturbance related to traditional ED pathology and over-evaluation of weight/shape is referred to as ‘disordered eating’. While most research on atypical eating in autism has been conducted with children, autism continues to affect eating behaviours into adulthood, with these behaviours possibly contributing to unhealthily high or low body weight (Kinnaird et al., 2019). High prevalence of autism in ED populations may be associated with increased rates of atypical eating, but not disordered eating, which, in the absence of sensitive and specific ED diagnostic tools, leads to false characterisation of some autistic adults.

The inclusion of avoidant/restrictive food intake disorder (ARFID) in the latest Diagnostic and Statistical Manual, Fifth Edition (DSM-5; APA, 2013) could aid with differential diagnosis, but research on ARFID in autism is in its infancy (Lucarelli et al., 2017). While ARFID may characterise some aspects of atypical eating, other ritualistic or rule-based eating patterns (e.g., selective eating) may not involve restricted eating. In fact, autistic individuals are at increased risk of being overweight and obese as well as underweight (Phillips et al., 2014; Sedgewick et al., 2020). Inclusion of Binge Eating Disorder (BED) in DSM-5, characterised by episodes of binge eating in the absence of compensatory behaviours, has also sparked debate over whether the core psychopathology of EDs (i.e., over-evaluation of weight/shape) should be present in all ED diagnoses (Grilo, 2013; Grilo et al., 2010). Spek et al. (2019) used the Swedish Eating Assessment for Autism Spectrum Disorders (SWEAA; Karlsson et al., 2013) to compare atypical eating in autistic and non-autistic adults. Autistic adults, particularly women, reported various forms of atypical eating, including eating rituals, sensory sensitivity, and social difficulties with eating, as well as disordered eating (ED pathology). ED symptoms reported by these participants may have been driven by atypical eating, rather than by weight/shape concern. Thus, examining whether atypical eating is associated with traditional ED pathology (driven by weight/shape concern) in autistic adults will help address this issue.

Interest in the relationship between autism and EDs has also arisen due to similarities in cognitive, social and emotional difficulties in the two populations (Davies et al., 2016; Oldershaw et al., 2011; Westwood et al., 2016b; Westwood, Stahl, Westwood et al., 2016a, b, c). One factor that may explain socio-emotional difficulties seen in autism and EDs is alexithymia (difficulties describing and identifying one’s own emotions; Nemiah, 1976), which co-occurs with both EDs and autism (Kinnaird, Stewart et al., 2019; Westwood et al., 2017). Emotional difficulties have been implicated in theoretical models of EDs, which have posited that disordered eating arises from a need to manage intolerable or unacceptable emotions (Cooper, 2005; Fairburn & Harrison, 2003), which may be associated with alexithymia (Nowakowski et al., 2013). While specific research on the contribution of alexithymia to the development of EDs is scarce (Nowakowski et al., 2013), alexithymia predicts poor recognition of others’ emotion in autism (Cook et al., 2013) and EDs (Brewer et al., 2015). Hobson et al. (2020) found that alexithymia explained the likelihood of meeting ADOS criteria for autism in women with AN, meaning individuals with EDs could score highly on autism assessments because they tap into multiple constructs (e.g., co-occurring alexithymia).

Another common feature of both autism and EDs, which is closely related to alexithymia (Murphy et al., 2018b), is atypical interoception; one’s perception of internal states, such as cardiac, respiratory and gastric signals (Craig, 2002; Khalsa & Lapidus, 2016). A multi-factorial model distinguishing between interoceptive accuracy (the ability to accurately perceive interoceptive signals), and attention (the propensity to attend to interoceptive signals), has been proposed (Murphy et al., 2018b, 2019) and supported with empirical research (Murphy, Brewer, Murphy et al., 2018a, b). Models also distinguish between objective performance measures of interoception, and subjective beliefs about one’s interoceptive abilities (Garfinkel et al., 2016; Murphy et al., 2018b). The majority of objective tasks assess interoceptive accuracy (e.g. cardiac perception; Schandry, 1981), but self-report measures can separately measure interoceptive attention (e.g., the Body Perception Questionnaire (BPQ); Porges, 1993) and interoceptive accuracy (e.g., the Interoceptive Accuracy Scale (IAS); Murphy, Brewer, Murphy et al., 2018a, b).

While autism has been theorised to be characterised by impaired interoception (Quattrocki & Friston, 2014), empirical research has produced mixed findings. Different studies have found reduced (e.g., DuBois et al., 2016; Fiene & Brownlow, 2015) and increased (Garfinkel et al., 2016) self-reported interoceptive attention in autism. Similarly, evidence for reduced interoceptive accuracy in autism has been mixed (Hatfield et al., 2019). It is also unclear how interoception is related to behavioural characteristics in autism (DuBois et al., 2016). Brewer et al. (2015) argue that alexithymia, not autism, is the consequence of interoceptive atypicalities. Alexithymia co-occurs with autism (Kinnaird, Stewart et al., 2019), and many of the features noted by Quattrocki and Friston to be common in autism and likely to depend on interoception (e.g., poor eye contact, empathy, and emotion recognition) are not present in the absence of alexithymia (Bird et al., 2010, 2011; Cook et al., 2013; Oakley et al., 2016). Notably, alexithymia and interoception are still distinctly defined, with alexithymia representing interoceptive difficulties specifically in the emotional domain. If atypical interoception gives rise to alexithymia, which in turn explains the socio-emotional difficulties observed in both autism and EDs, then interoception could explain some of the disordered eating observed in autism, through the mediating effect of alexithymia. Alternatively, alexithymia and interoception could be linked to atypical/disordered eating through distinct pathways, the former by disordered eating functioning to manage emotional difficulties and the latter due to problems perceiving hunger or satiety signals.

Atypical interoception has been observed in EDs (Khalsa & Lapidus, 2016), although findings are inconsistent. Several studies have found that reduced interoceptive attention is related to disordered eating (e.g., Fassino et al., 2004), although dietary restraint may be associated with non-acceptance of internal states, rather than lack of clarity (Merwin et al., 2010). Others have shown no difference in cardiac interoceptive accuracy (e.g., Eshkevari et al., 2014). Self-reported interoceptive accuracy has been measured extensively in EDs using the Eating Disorder Inventory (EDI; Garner et al., 1983), with a meta-analysis reporting substantial interoceptive deficits across multiple EDs (Jenkinson et al., 2018). Autistic women have also reported difficulties identifying hunger as a contributing factor to their restrictive ED in qualitative interviews (Brede et al., 2020). A recent study found that cardiac interoceptive accuracy was typical in AN, but confidence in task performance was significantly lower (Kinnaird et al., 2020). Interoception was not associated with autistic traits or alexithymia.

Atypical eating may also be associated with atypical interoception (Herbert & Pollatos, 2012). In Kinnaird et al. (2019a, b, c) qualitative study, autistic women with AN commented that sensory difficulties and compulsive exercise as a method of stimulation, possibly due to inducing changes in muscle tension, heart rate and breathing, contributed to their EDs. It is therefore possible that interoception contributes to atypical eating, and in turn to ED pathology, in autistic individuals. Indeed, interoceptive accuracy fully mediates the relationship between intuitive eating (eating in response to hunger) and BMI, suggesting interoception is responsible for intuitive eating (Herbert et al., 2013). As atypical interoception is elevated in autism (Quattrocki & Friston, 2014), interoception may explain high rates of atypical eating observed in autism.

**Fig. 1:**
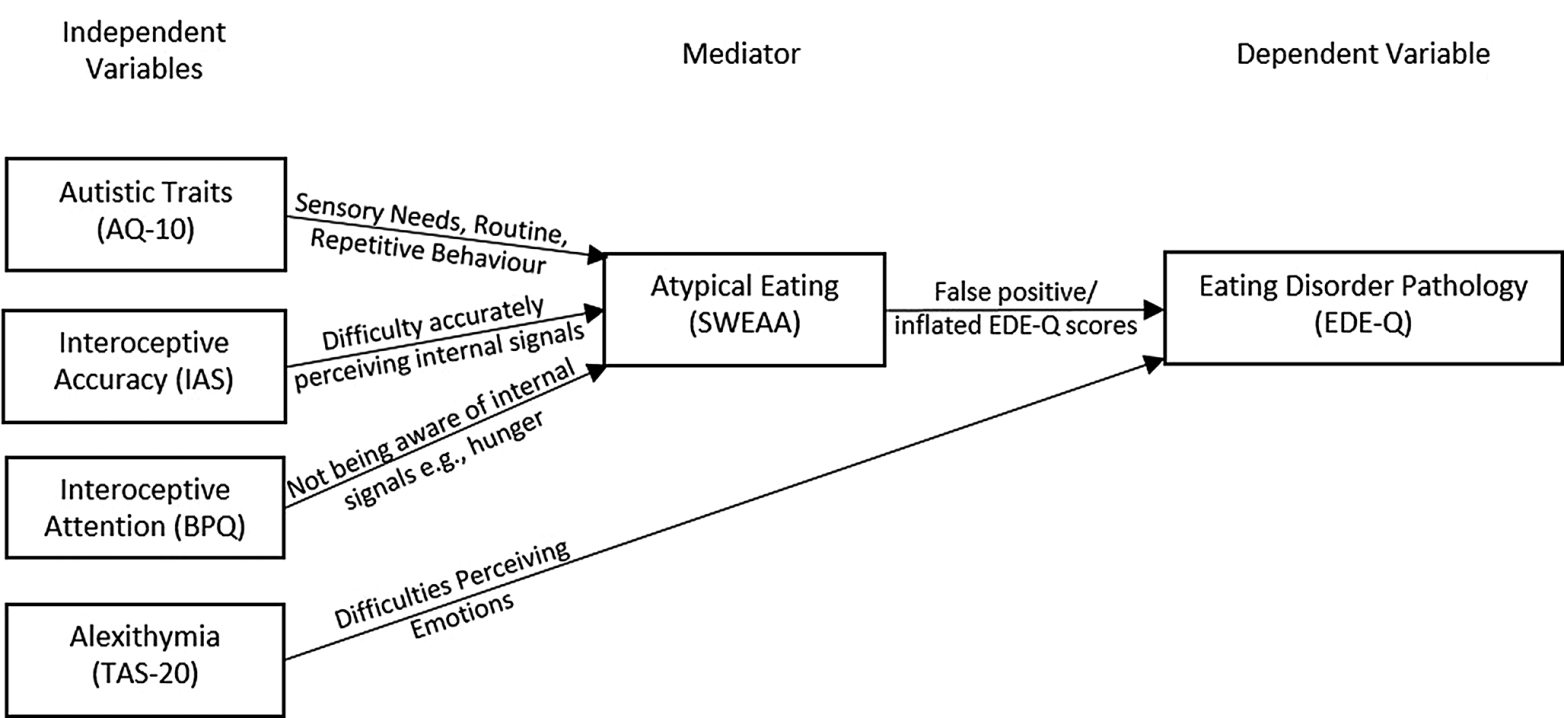
Proposed relationship between autistic traits, interoception, alexithymia and eating in autistic adults

This study investigated whether ED pathology is associated with atypical eating and other factors associated with EDs and autism, namely interoceptive accuracy and attention, alexithymia, and autistic traits. The hypothesised relationship between these variables in autistic individuals is shown in Fig. 1. Structural Equation modelling was used to compare the relationship between these traits, atypical eating and ED pathology in autistic and non-autistic adults, to determine whether eating difficulties experienced by these groups are distinct in nature.

It was hypothesised that autism (diagnostic group) would moderate the relationship between interoception, alexithymia, autistic traits and disordered eating (H_1_) whereby exogenous variables in the model would account for a significantly higher proportion of the variance in ED pathology in the autistic than non-autistic group (as, relative to the traditional over-evaluation of weight/shape factors, these are expected to be more important contributors in autism than the non-autistic population). Additionally, it was hypothesised that the positive relationship between atypical eating and ED pathology would be stronger in autistic than non-autistic adults (H_2_) and the indirect effect of autistic traits, alexithymia and interoception on ED pathology (through atypical eating) would be stronger for autistic than non-autistic adults (H_3_).

Finally, it was hypothesised that the strength of the relationship between body image (a key contributor to traditional ED pathology) and disordered eating would be stronger in non-autistic than autistic adults (H_4_).

## Method

## Participants

191 autistic and 206 non-autistic adults took part in this study. Autistic adults were recruited through social media and Autistica. Non-autistic adults were recruited through social media and the Royal Holloway University of London Psychology volunteer recruitment system and thus represented students and those in the general population with an interest in the topic. Autistic participants were required to have a formal diagnosis of autism. Participants self-reported diagnosis and provided details of the diagnostic process (e.g., where they were diagnosed, health care professionals involved, specific diagnosis given). On the AQ-10, 92.1% of autistic and 15% of non-autistic participants scored at or above the recommended cut-off of six, suggesting self-reported diagnosis was reliable.

The autistic group was significantly older, *M* = 37.49 years, *SD* = 12.55, than the non-autistic group, M = 28.39, *SD* = 10.71, *t*(375) = 7.74, *p* <.001, and reported higher rates of current/previous ED diagnoses, χ^2^(1)^=^22.10, *p* <.001, and psychiatric diagnoses in general, χ^2^(1)^=^91.93, *p* <.001. As the study description mentioned autism and eating, self-selection bias may have led to over-representation of EDs in both samples. There was no difference in sex between the two groups, χ^2^(1)^=^0.14, *p* = .712, but median BMI was higher in the autistic (23.74) than non-autistic (22.22) group, *U* = 15,624, z=-2.44, *p* = .011. More autistic individuals met criteria for being both over and underweight than the non-autistic group. Demographic information for each group is displayed in Supplementary Material 1.

A-priori sample size was calculated based on needing ten times as many observations as variables (Hair et al., 2010). As there were eight variables in the final model, a sample of > 80 per group was required. Ethical approval for the study was granted by the local research ethics committee.

### Measures

ED symptomology was measured by the Eating Disorder Examination Questionnaire (EDE-Q; Fairburn & Beglin, 1994), which yields a ‘global’ score and four subscale scores (eating concern; weight concern; shape concern and restriction) with higher scores indicating more severe ED symptoms.

The Swedish Eating Assessment for Autism Spectrum Disorders (SWEAA; Karlsson et al., 2013) assessed atypical eating. The SWEAA is a self-report questionnaire measuring eating and mealtime problems, validated for use in autistic individuals. It consists of 60 items with 10 subscales: perception; motor control; purchase of food; eating behaviour; mealtime surroundings; social situation at mealtimes; other behaviours associated with disturbed eating; hunger/satiety; simultaneous capacity, and Pica, with higher scores indicating more eating difficulties. As there is some overlap between SWEAA subscale scores and other constructs included in the proposed model (e.g., ED pathology and interoception), a latent variable was constructed using only the subscales of the SWEAA that did not overlap theoretically with other variables, to reduce shared variance. The following subscales were removed prior to exploratory or confirmatory factor analysis (CFA) procedures: perception, motor control, and hunger/satiety (all closely related to interoception), other behaviours associated with disturbed eating (which reflects ED pathology), and pica (which was not relevant to the study’s objectives). Cronbach’s alpha for the reduced scale used in this study was 0.94.

Weight and body image concerns were assessed with the Body Uneasiness Test, Part A (BUT-A; Cuzzolaro et al., 2006), to test the association between ED pathology and body image in autistic and non-autistic individuals, with higher scores indicating greater body image problems. It was not included in the final structural equation model due to the expected high level of shared variance with the EDE-Q owing to overlap in theoretical constructs.

Alexithymia was assessed using the Twenty-item Toronto Alexithymia Scale (TAS-20; Bagby, Parker et al., 1994), with higher scores indicating greater levels of difficulty (Taylor et al., 1999).

Interoceptive attention was assessed with the Body Perception Questionnaire, Awareness subscale (BPQ; Porges, 1993), with higher scores indicating greater interoceptive attention.

Interoceptive accuracy was assessed with the Interoceptive Accuracy Scale (IAS; Murphy et al., 2018), with higher scores indicating greater perceived interoceptive accuracy.

The Autism Spectrum Quotient, short version (AQ-10; Allison et al., 2012) was used categorically to support self-reported autism diagnosis, and as a continuous measure of autistic traits within the final structural model.

Mood over the last week was assessed using the 21-item Depression, Anxiety and Stress Scale (DASS-21; Lovibond & Lovibond, 1995), with higher scores indicating greater emotional difficulties. The DASS-21 was used to control for depression, anxiety and stress in analyses. The three subscale scores were used to create a latent variable, entitled “mood” through exploratory and confirmatory factor analysis, as outlined in Supplementary Material 1.

### Procedure

All measures were completed online using Qualtrics (Provo, UT) in the following order: demographic information, DASS21, TAS-20, EDE-Q, IAS, SWEAA, AQ-10, BUT-A, BPQ.

## Results

Data were checked for normality visually. Shapiro-Wilks tests were not used due to being overly conservative with large samples (Ghasemi & Zahediasl, 2012). If distributions were not normal across both groups, both parametric and non-parametric tests were performed. Where both tests were significant (*α* = 0.0045; Bonferroni corrected), parametric results are reported.

Group mean scores and Bonferroni corrected independent samples *t*-tests across the autistic and non-autistic samples are displayed in Table 1. The autistic group scored significantly higher on all measures except for the IAS, on which they scored significantly lower than the non-autistic group. As age differed between the two groups, age was included as a covariate in subsequent analysis and in the structural model.

**Table 1:** Comparison of autistic and non-autistic groups on self-report measures

	Autistic (*n* = 191) mean (SD)	Non-autistic (*n* = 206) mean (SD)	p**	Cohen’s d effect size
EDE-Q Global	2.65(1.65)	1.81(1.36)	< 0.001	0.55
Atypical Eating	46.07(17.35)	24.58(14.47)	< 0.001	1.35
BUT-A	1.93(1.08)	1.25(0.95)	<. 001	0.67
IAS	71.08(13.86)	82.15(11.41)	<. 001	0.87
TAS-20	65.31(12.61)	48.58(12.93)	< 0.001	1.31
BPQ	131.25(32.64)	112.09(39.69)	< 0.001	0.53
AQ-10	8.10(1.74)	3.07(2.40)	< 0.001	2.40
DASS21 Anxiety Depression Stress	14.07(9.76) 17.84(11.77) 23.06(10.61)	7.07(7.70) 9.73(10.38) 13.94 (9.75)	< 0.001 < 0.001 < 0.001	0.80 0.73 0.90

**Significance level set at 0.0045 to account for multiple comparisons

Across the whole sample, there was a significant strong positive correlation between BUT-A and EDE-Q global scores, *r*(395) = 0.83. *p* <.001. Fisher’s r-to-z and Potthoff analysis indicated that this relationship was comparable across the autistic and non-autistic groups in terms of strength (*Z* = 1.34, *p* = .090) and slope (*F*_3,389=_285.23, *p* = .280). EDE-Q scores therefore appear to assess shape and weight concerns effectively in both groups, contrary to H_4_.

**Fig. 2:**
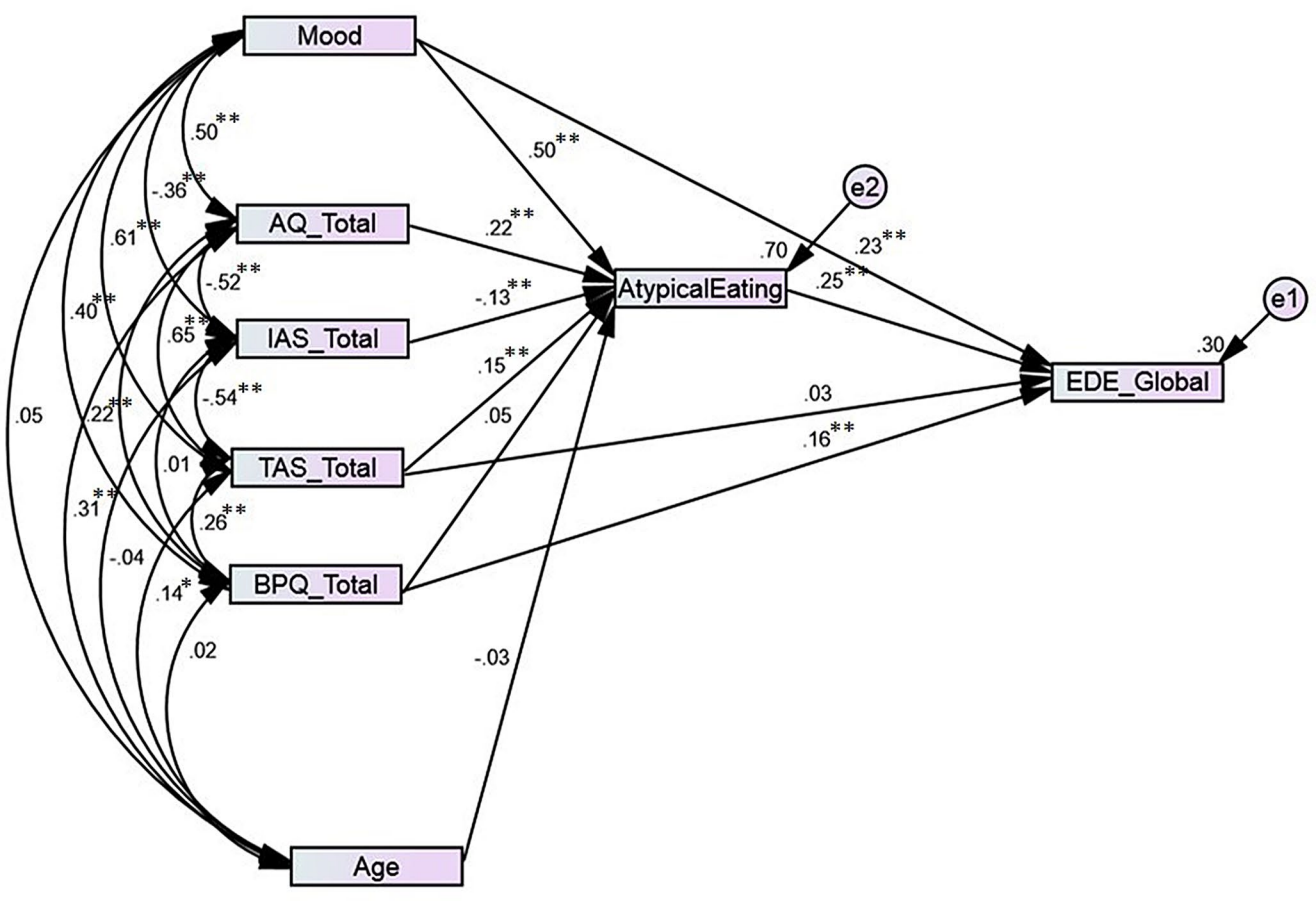
Final mediated structural model for the entire sample. Total *n* = 397. Rectangles represent observed variables, with atypical eating and mood representing composite variables from the latent factor scores. Single headed and double headed arrows represent the impact of one variable on another and correlation between pairs of variables, respectively. ** *p* <.001; *p <.01

The structural model for the entire sample (created using Amos; Byrne, 2016), with R-squared and beta coefficients, is displayed in Fig. 2. Analyses relating to the pre-requisites of SEM are presented in Supplementary Material 1. There were significant positive associations between mood and EDE-Q global scores, mood and atypical eating, AQ-10 scores and atypical eating, TAS-20 and both atypical eating and EDE-Q scores, and between BPQ and both atypical eating and EDE-Q scores. There was a significant negative association between IAS scores and atypical eating. Age was not significantly associated with atypical eating, but was significantly positively correlated with TAS-20 and AQ-10 scores. Bivariate correlations between the variables included in this model are shown in Supplementary Material.

Initial evaluation of the structural model suggested a good fit of the data (see Supplementary Material), indicating that atypical eating played a mediating role. Across the entire sample, the positive indirect effects from AQ-10 scores to EDE-Q scores (*β* = 0.054, 95% CI [0.024, 0.097], *p* = .002) and from TAS-20 to EDE-Q scores (*β* = 0.037, 95% CI [0.014, 0.079], *p* = .003), and the negative indirect effect from IAS scores to EDE-Q scores (*β=*-0.031, 95% CI [.-0.066, − 0.010], *p* = .004) via atypical eating were significant. The indirect effect from BPQ to EDE-Q scores was not significant, indicating no mediating effect of atypical eating on this relationship.

**Fig. 3:**
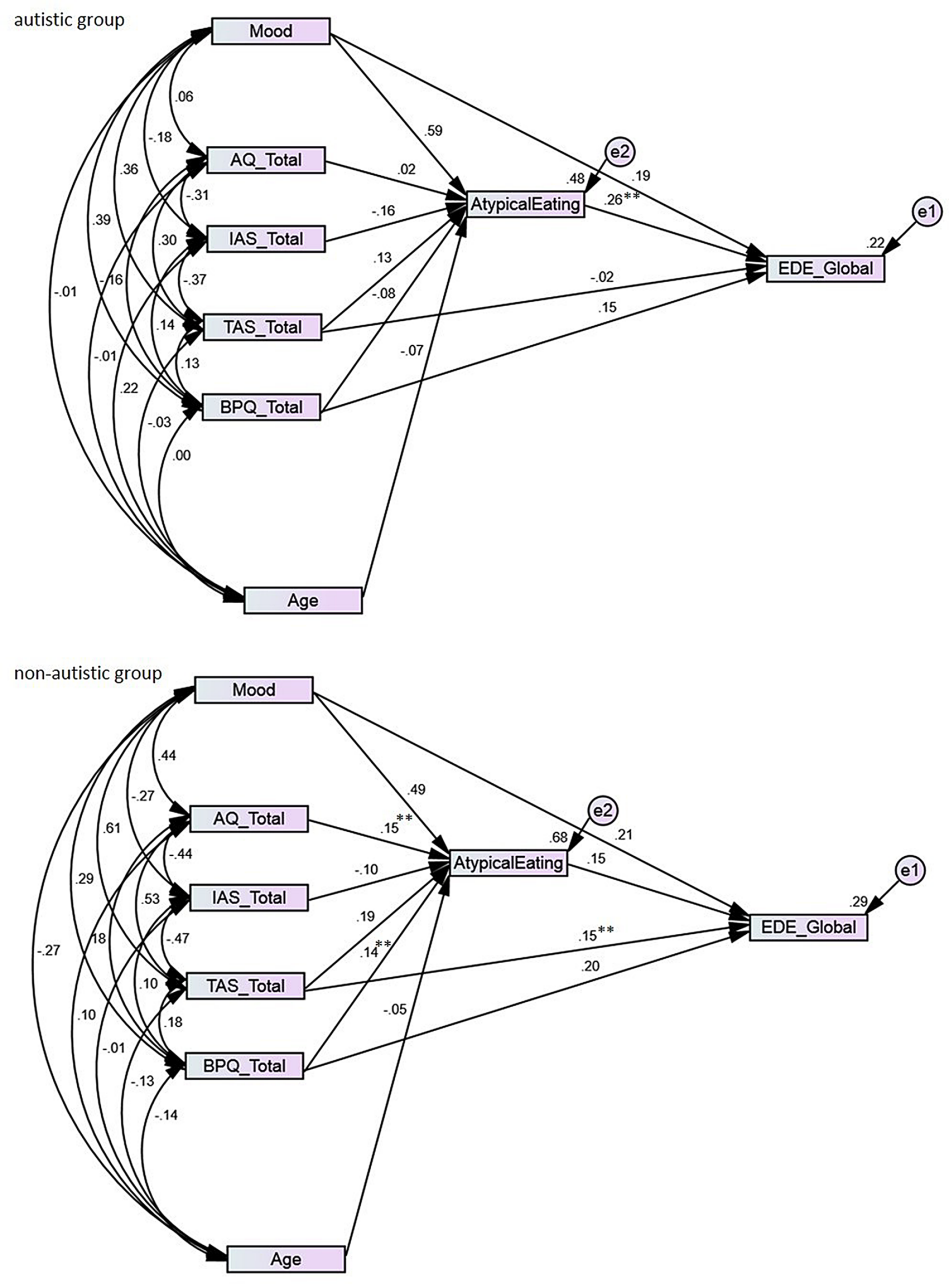
Mediated models for the autistic group (top), *n* = 191, and non-autistic group, *n* = 206. Rectangles represent observed variables, with atypical eating and mood representing composite variables from the latent factor scores. Single headed and double headed arrows represent the impact of one variable on another and correlation between pairs of variables, respectively. AQ_Total = total score of the ten-item autism-spectrum quotient; BPQ_Total = total score of the Body Perception Questionnaire, awareness section; EDE_Global = global score of the Eating Disorder Examination Questionnaire; IAS_Total = total score of the Interoceptive Accuracy Scale; TAS_Total = total score of the 20-item Toronto Alexithymia Scale. **=relationship was only significant in that group

To determine whether there was a global difference in the structural model between groups, multi-group moderation compared the fit of the structural model for autistic and non-autistic groups separately. Moderated models in which the parameters were freely estimated and constrained to equality across groups were compared with chi-square difference tests. Standardised regression coefficients and R-squared values for each group, in which the structural path’s values were constrained to be equal for the two groups, are displayed in Fig. 3. Multi-group analysis revealed no difference at the full structural model level between the autistic and non-autistic groups (Δχ^2^(10) = 12.26, *p* = .268), contrary to hypothesised (H_1_). Local tests examining specific pathway differences between the groups were observed, although these should be interpreted with caution considering the lack of group difference in the full model. The relationships between AQ-10 scores and atypical eating, BPQ scores and atypical eating, and TAS-20 scores and EDE-Q global scores were only significant in the non-autistic group. Further, the standardised regression coefficient for the positive relationship between atypical eating and EDE-Q scores was only significant for the autistic group (*β =* 0.265, *p* <.010, ΔZ = 0.012), suggesting some moderation by group, in line with H_2_.

To test whether the indirect effects of autistic traits, alexithymia and interoception on ED pathology (through atypical eating) were stronger for autistic adults than non-autistic adults (H_3_), it was first necessary to determine whether atypical eating mediated these relationships in the entire sample (global model; see Supplementary Material). Comparison of the mediating effect of atypical eating between the autistic and non-autistic groups showed no significant difference in the indirect effect of AQ-10 (*β =* 0.009, 95% CI [-0.024, 0.050], *p* = .546), IAS (*β =* 0.003, 95% CI [-0.003, 0.010], *p* = .260) or TAS-20 scores (*β=*-0.001, 95% CI [-0.009, 0.005], *p* = .643), contrary to hypothesised (H_3_).

### Supplementary Analysis

To test the robustness of the mediated model and in line with SEM recommendations (e.g., Weston & Gore Jr, 2006), the structural model was compared with two alternative theoretical models for the relationship between the variables (see Supplementary Material).

## Discussion

This study investigated whether eating difficulties experienced by autistic adults are distinct in their underlying pathology, by examining the extent to which atypical eating (eating disturbance not related to weight/shape concerns) mediates the relationship between ED pathology and interoception, alexithymia and autistic traits, in both autistic and non-autistic samples. The overall model was a good fit for the data, but group did not moderate these relationships. Further, body image dissatisfaction was similarly related to ED pathology in both groups. However, atypical eating only significantly predicted ED pathology in the autistic group, while autistic traits, interoceptive attention and alexithymia were only predictive of ED pathology in the non-autistic group, suggesting that individual predictors of disordered eating do vary as a function of autistic status.

The autistic group self-reported significantly higher ED pathology, body image dissatisfaction, mood difficulties, alexithymia and interoceptive attention, and significantly lower interoceptive accuracy than the neurotypical group. The elevated ED pathology and body image dissatisfaction observed in the autistic group support the notion that autism is associated with increased risk of EDs, consistent with previous research showing over-representation of autism in EDs (Westwood & Tchanturia, 2017) and elevated ED pathology in autism (Kalyva, 2009). The autistic group also reported significantly higher rates of current or previous ED diagnoses, consistent with a nationwide register-based cohort study, which found that autistic probands had an increased risk of developing AN (Koch et al., 2015). In the current study, self-reported psychiatric diagnoses, and depression, anxiety and stress scores, were generally elevated in the autistic group. Koch and colleagues found the risk of autistic individuals developing AN was even higher following a diagnosis of depression, suggesting that the relationship between autism and AN may be non-specific. Alternatively, additive effects of autism and depression may exacerbate atypical or disordered eating. In the final structural model for the entire sample, however, the significant association between mood and EDE-Q scores did not differ by group, suggesting depression may not be of increased importance in the treatment of autistic adults with EDs.

Unsurprisingly, alexithymia was higher in autistic than non-autistic participants (for a review, see Kinnaird, Stewart et al., 2019). That the autistic group self-reported higher interoceptive attention but lower interoceptive accuracy also replicates previous research (Garfinkel et al., 2016). Notably, the same pattern of high interoceptive attention but low interoceptive accuracy has been observed in alexithymia (Betka et al., 2018; Ernst et al., 2014), adding strength to the theory that alexithymia, not autism, is the consequence of atypical interoception (Brewer et al., 2015). While interoceptive attention may be increased to compensate for low perceived interoceptive accuracy, findings in autistic samples have varied (DuBois et al., 2016; Fiene & Brownlow, 2015; Schauder et al., 2015), with further research into this relationship required.

The hypothesis that the strength of the relationship between ED pathology and body image dissatisfaction would be weaker in the autistic group was not supported. While it is possible that atypical eating falsely inflates scores on ED measures such as the EDE-Q, the current results suggest that weight/shape concern is still associated with ED pathology in autistic adults. Although autistic women with AN have cited concerns with weight/shape as less relevant in the development of their ED (Kinnaird, Norton, Stewart, et al., 2019), these could still have been a contributing factor. They might have different underlying causes to these concerns in non-autistic individuals, however. Indeed, Brede et al. (2020) interviewed autistic participants who reported shape and weight concerns following ED treatment, due to imitating women they met during ED treatment, perhaps as a form of camouflaging. Additionally, the factors responsible for the unexplained variance in EDE-Q scores may differ between the groups (e.g. need for routine or following specific rules in the autistic group).

While the overall model was invariant across groups, the direct relationship between atypical eating (not driven by weight/shape) and ED pathology was only significant in the autistic group. Therefore, while body image appears to contribute to ED pathology in autistic adults, atypical eating may represent an additional unique risk factor for ED pathology. While the correlational nature of this study precludes inferences about causality, the combination of atypical eating and body image dissatisfaction may partially account for elevated ED pathology in autism. The limited research that has examined the nature of atypical eating in autistic individuals (e.g., Spek et al., 2019) reported that autistic women experienced eating problems unrelated to ED pathology, such as sensory sensitivity and social difficulties with eating, as well as traditional ED characteristics.

The positive relationship between interoceptive attention and atypical eating was only significant in the non-autistic group. While interoceptive attention has been related to intuitive eating and aspects of ED pathology (Fassino et al., 2004), and may mediate the relationship between body appreciation and intuitive eating (Oswald et al., 2017), its relationship with other types of atypical eating lacks investigation. While group differences may be explained by lower variance in BPQ scores within the autistic group, it seems likely that the high rates of atypical eating in the autistic group were driven by factors unrelated to interoceptive attention, such as need for routine and sameness, and difficulties eating in social contexts. If these causes of atypical eating are less common in the non-autistic population, the role of interoceptive attention in atypical eating may be exaggerated in the non-autistic relative to autistic group. Further research is needed, however, to examine the multiple potential routes to atypical eating across populations.

Lower self-reported interoceptive accuracy was associated with higher atypical eating behaviour across the entire sample. Difficulties perceiving hunger and satiety signals likely make it difficult to use these signals to inform one’s eating, consistent with findings that interoceptive accuracy is positively associated with intuitive eating (Herbert et al., 2013). In the current study the relationship between interoceptive accuracy and ED pathology was mediated by atypical eating in both groups, suggesting that it is unusual eating behaviour in general, and not ED pathology concerned with weight/shape specifically, that is impacted by interoception.

Atypical eating also mediated the positive relationship between alexithymia and ED pathology in the whole sample, although when parameters were constrained to equality across groups, this path was not significant in either group. While alexithymia is known to be common in autism (Kinnaird, Stewart et al., 2019) and EDs (Westwood et al., 2017), these results suggest that alexithymia explains a similar proportion of the variance in ED pathology in autistic and non-autistic individuals. The hypothesis that alexithymia would account for more ED variance in the autistic group (H_1_) was based on theories proposing that EDs develop as a way of managing/suppressing unacceptable and/or frightening emotions, by controlling eating (Cooper, 2005; Fairburn et al., 2003). Interestingly, the direct relationship between TAS-20 and EDE-Q scores in this study was not significant, suggesting that atypical eating fully mediates this relationship. Previous work suggests that the relationship between alexithymia and ED pathology is also mediated by clinical perfectionism (Marsero et al., 2011), supporting the idea that this relationship is indirect. This relationship might vary as a function of sex, however, with recent work finding that alexithymia directly mediated the relationship between autistic traits and ED symptomatology in women, while in both women and men this relationship was also mediated via alexithymia and then depressive and anxious symptoms sequentially (Moseley et al., 2024).

Finally, across the whole sample, atypical eating mediated the positive relationship between AQ-10 and EDE-Q scores, but the relationship between AQ-10 and atypical eating was only significant in the non-autistic group. As in the case of alexithymia, the mediating effect of atypical eating on EDE-Q scores suggests that rather than there being a direct link between autistic traits and specific ED pathology, it could be that such traits increase disturbances in eating in general, e.g. due to rigidity or obsessionality (Kinnaird, Norton, Stewart et al., 2019), which inflate scores on measures of ED pathology. While firm conclusions about causality cannot be drawn from correlational studies, this finding raises the possibility that the relationship between ED pathology and interoception, alexithymia or autistic traits is not direct. Alternatively, the non-significant relationship between autistic traits and atypical eating in the autistic group may be due to lower variance in AQ-10 scores, as most autistic participants scored highly on this measure. As the AQ-10 was designed as a brief screening tool (Booth et al., 2013), it may also be less valid as a measure of individual differences (Taylor et al., 2020).

Taken together, the findings from this study provide some evidence that the nature of ED pathology is different in autistic and non-autistic adults, with implications for treatment. Previous research has concluded that autistic people within ED services are likely to have more complex needs than non-autistic individuals (Babb et al., 2021; Babb et al., 2022; Nazar et al., 2018; Stewart et al., 2017; Tchanturia et al., 2017). If, as suggested by the current study, ED pathology in autistic adults is related to both atypical eating and weight/shape concerns, diagnosis should consider the presence of eating disturbances not related to the core psychopathology of EDs, and ED treatment should consider autistic traits, e.g. by respecting food preferences. Indeed, participants in Kinnaird, Norton, Stewart, et al.‘s (2019) study reported wanting treatment to recognise and address eating issues associated with autism.

The current study also indicates that weight/shape concerns should not be overlooked in autistic adults, and ED assessment should consider the full range of social, emotional and cognitive issues which may contribute to ED pathology in this population. While EDs in autistic individuals may still stem from weight/shape concerns, however, such concerns may arise for different reasons (e.g., being encouraged by health professionals to lose weight, bullying over weight/shape, internalisation of the thin ideal due to camouflaging).

The current findings should be interpreted in the context of some limitations. Although the sample was large, data were based on self-report, including diagnostic information. Self-reported interoceptive attention should also be interpreted cautiously, as interpretation of BPQ questions appears to vary amongst participants (Gabriele et al., 2021).The small number (16) of non-autistic participants reporting an ED diagnosis precluded any analysis of interactions between autism and ED diagnosis, and while the autistic and non-autistic groups were well matched on sex, most participants were female, which is unrepresentative of the autistic population (Lai et al., 2015). Data on behavioural characteristics of the groups (e.g., IQ), were not collected. Autism is a spectrum condition with wide variation in the profile of features, and thus potential interactions between factors such as intelligence, severity and eating pathology cannot be ruled out. Further, the results may be less relevant to those with high severity of core autistic symptoms, co-occurring intellectual disability, little or no language and requiring extensive care (sometimes termed “profound autism”; Lord et al., 2022). It is possible that this subgroup would have fewer concerns about weight or shape, and would likely not have participated in the current study due to the requirement to complete self-report questionnaires.

The research subject may have been of particular interest to females, and those with eating disturbances, potentially accounting for the high proportion of females and those with ED diagnoses, and for some of the observed group differences, although autism and EDs are known to co-occur (Westwood & Tchanturia, 2017). Correspondingly, as the non-autistic group was recruited via the university Psychology volunteer recruitment service and social media, participants may have had a particular interest in this topic. This may have led to a higher prevalence of psychiatric co-morbidity than would be observed in the general population. Further, as weight and autism status are confounded in this sample (and the population; McCoy et al., 2016; Phillips et al., 2014; Sedgewick et al., 2020), with more autistic than non-autistic participants meeting the overweight criteria, high autistic EDE-Q scores may have been partially driven by appropriate weight loss strategies. ED measures in autism may require adaptation to distinguish between healthy and unhealthy weight loss behaviours.

In conclusion, this is the first study to investigate factors associated with atypical eating and ED pathology, including interoception and alexithymia, in autistic and non-autistic adults. Results suggested that the proposed model of the relationship between these variables did not differ by group, but the relationship between atypical eating and ED pathology was only significant in the autistic group. However, the strength of the relationship between body image and ED pathology was comparable across groups, indicating that body image dissatisfaction does play a role in ED pathology in autistic adults. Treatment approaches that consider the multi-faceted nature of EDs in autistic individuals are therefore warranted.

## Electronic supplementary material

Below is the link to the electronic supplementary material.

Supplementary Material 1

## Data Availability

Data are available at https://osf.io/8z26m/.

## References

[CR1] Allison, C., Auyeung, B., & Baron-Cohen, S. (2012). Toward brief red flags for autism screening: The short Autism Spectrum Quotient and the short quantitative checklist for Autism in toddlers in 1,000 cases and 3,000 controls [corrected]. *Journal of the American Academy of Child and Adolescent Psychiatry*, *51*(2), 202–212e207. 10.1016/j.jaac.2011.11.003 22265366 10.1016/j.jaac.2011.11.003

[CR2] APA. (2013). *Diagnostic and statistical manual of mental disorders* (5th ed.).). American Psychiatric Publishing.

[CR3] Bagby, R. M., Parker, J. D., & Taylor, G. J. (1994a). The twenty-item Toronto Alexithymia Scale–I. item selection and cross-validation of the factor structure. *Journal of Psychosomatic Research*, *38*(1), 23–32. http://www.ncbi.nlm.nih.gov/pubmed/8126686 8126686 10.1016/0022-3999(94)90005-1

[CR4] Bagby, R. M., Taylor, G. J., & Parker, J. D. (1994b). The twenty-item Toronto Alexithymia Scale–II. Convergent, discriminant, and concurrent validity. *Journal of Psychosomatic Research*, *38*(1), 33–40. http://www.ncbi.nlm.nih.gov/pubmed/8126688 8126688 10.1016/0022-3999(94)90006-x

[CR6] Bentz, M., Jepsen, J. R. M., Pedersen, T., Bulik, C. M., Pedersen, L., Pagsberg, A. K., & Plessen, K. J. (2017). Impairment of social function in young females with recent-onset Anorexia Nervosa and recovered individuals. *Journal of Adolescent Health*, *60*(1), 23–32. 10.1016/j.jadohealth.2016.08.011 28341015

[CR101] Betka, S., Pfeifer, G., Garfinkel, S., Prins, H., Bond, R., Sequeira, H., Duka, T., & Critchley, H. (2018). How do self-assessment of alexithymia and sensitivity to bodily sensations relate to alcohol consumption? *Alcoholism: Clinical and Experimental Research, 42*(1), 81–88. 10.1111/acer.13542 29094768

[CR8] Bird, G., Silani, G., Brindley, R., White, S., Frith, U., & Singer, T. (2010). Empathic brain responses in insula are modulated by levels of alexithymia but not autism. *Brain*, *133*(5), 1515–1525. 20371509 10.1093/brain/awq060 PMC2859151

[CR7] Bird, G., Press, C., & Richardson, D. C. (2011). The role of alexithymia in reduced eye-fixation in autism spectrum conditions. *Journal of Autism and Developmental Disorders*, *41*(11), 1556–1564. 21298331 10.1007/s10803-011-1183-3

[CR9] Blinder, B. J., Cumella, E. J., & Sanathara, V. A. (2006). Psychiatric comorbidities of female inpatients with eating disorders. *Psychosomatic Medicine*, *68*(3), 454–462. 10.1097/01.psy.0000221254.77675.f5 16738079 10.1097/01.psy.0000221254.77675.f5

[CR10] Booth, T., Murray, A. L., McKenzie, K., Kuenssberg, R., O’Donnell, M., & Burnett, H. (2013). Brief report: An evaluation of the AQ-10 as a brief screening instrument for ASD in adults. *Journal of Autism and Developmental Disorders*, *43*(12), 2997–3000. 10.1007/s10803-013-1844-5 23640304 10.1007/s10803-013-1844-5

[CR908] Brede, J., Babb, C., Jones, C., Elliott, M., Zanker, C., Tchanturia, K., Serpell, L., Fox, J., & Mandy, W. (2020). “For me, the anorexia is just a symptom, and the cause is the autism”: Investigating restrictive eating disorders in autistic women. *Journal of Autism and Developmental Disorders, 50*, 4280–4296. 10.1007/s10803-020-04479-3 PMC7677288 32274604

[CR11] Brewer, R., Cook, R., Cardi, V., Treasure, J., & Bird, G. (2015a). Emotion recognition deficits in eating disorders are explained by co-occurring alexithymia. *R Soc Open Sci*, *2*(1), 140382. 10.1098/rsos.140382 26064585 10.1098/rsos.140382 PMC4448790

[CR12] Brewer, R., Happé, F., Cook, R., & Bird, G. (2015b). Commentary on Autism, oxytocin and interoception: Alexithymia, not Autism Spectrum disorders, is the consequence of interoceptive failure. *Neuroscience and Biobehavioral Reviews*, *56*, 348–353. 10.1016/j.neubiorev.2015.07.006 26192103 10.1016/j.neubiorev.2015.07.006

[CR13] Byrne, B., M (2016). *Structural equation modeling with Amos: Basic concepts, applications and programming* (3rd ed.). Routledge.

[CR14] Castellini, G., Lo Sauro, C., Mannucci, E., Ravaldi, C., Rotella, C. M., Faravelli, C., & Ricca, V. (2011). Diagnostic crossover and outcome predictors in eating disorders according to DSM-IV and DSM-V proposed criteria: A 6-year follow-up study. *Psychosomatic Medicine*, *73*(3), 270–279. 10.1097/PSY.0b013e31820a1838 21257978 10.1097/PSY.0b013e31820a1838

[CR15] Cermak, S. A., Curtin, C., & Bandini, L. G. (2010). Food selectivity and sensory sensitivity in children with autism spectrum disorders. *Journal of the American Dietetic Association*, *110*(2), 238–246. 10.1016/j.jada.2009.10.032 20102851 10.1016/j.jada.2009.10.032 PMC3601920

[CR16] Cook, R., Brewer, R., Shah, P., & Bird, G. (2013). Alexithymia, not autism, predicts poor recognition of emotional facial expressions. *Psychological Science*, *24*(5), 723–732. 10.1177/0956797612463582 23528789 10.1177/0956797612463582

[CR17] Cooper, M. J. (2005). Cognitive theory in anorexia nervosa and bulimia nervosa: Progress, development and future directions. *Clinical Psychology Review*, *25*(4), 511–531. 15914267 10.1016/j.cpr.2005.01.003

[CR18] Craig, A. D. (2002). How do you feel? Interoception: The sense of the physiological condition of the body. *Nature Reviews Neuroscience*, *3*(8), 655–666. 12154366 10.1038/nrn894

[CR19] Cuzzolaro, M., Vetrone, G., Marano, G., & Garfinkel, P. (2006). The body uneasiness test (BUT): Development and validation of a new body image assessment scale. *Eating and Weight Disorders-Studies on Anorexia Bulimia and Obesity*, *11*(1), 1–13. 10.1007/BF03327738 16801740

[CR20] Davies, H., Wolz, I., Leppanen, J., Fernandez-Aranda, F., Schmidt, U., & Tchanturia, K. (2016). Facial expression to emotional stimuli in non-psychotic disorders: A systematic review and meta-analysis. *Neuroscience and Biobehavioral Reviews*, *64*, 252–271. 10.1016/j.neubiorev.2016.02.015 26915928 10.1016/j.neubiorev.2016.02.015

[CR21] DuBois, D., Ameis, S. H., Lai, M. C., Casanova, M. F., & Desarkar, P. (2016). Interoception in autism spectrum disorder: A review. *International Journal of Developmental Neuroscience*, *52*, 104–111. 27269967 10.1016/j.ijdevneu.2016.05.001

[CR22] Eddy, K. T., Dorer, D. J., Franko, D. L., Tahilani, K., Thompson-Brenner, H., & Herzog, D. B. (2008). Diagnostic crossover in anorexia nervosa and bulimia nervosa: Implications for DSM-V. *American Journal of Psychiatry*, *165*(2), 245–250. 18198267 10.1176/appi.ajp.2007.07060951 PMC3684068

[CR102] Ernst, J., Böker, H., Hättenschwiler, J., Schüpbach, D., Northoff, G., Seifritz, E., & Grimm, S. (2014). The association of interoceptive awareness and alexithymia with neurotransmitter concentrations in insula and anterior cingulate. *Social Cognitive and Affective Neuroscience, 9*(6), 857–863. 10.1093/scan/nst058 PMC4040102 23596189

[CR23] Eshkevari, E., Rieger, E., Musiat, P., & Treasure, J. (2014). An investigation of interoceptive sensitivity in eating disorders using a heartbeat detection task and a self-report measure. *European Eating Disorders Review*, *22*(5), 383–388. 24985151 10.1002/erv.2305

[CR24] Fairburn, C. G., & Beglin, S. J. (1994). Assessment of eating disorders - interview or self-report questionnaire. *International Journal of Eating Disorders*, *16*(4), 363–370://WOS:A1994PV44500004. 7866415

[CR26] Fairburn, C. G., & Harrison, P. J. (2003). Eating disorders. *The Lancet*, *361*(9355), 407–416. 10.1016/S0140-6736(03)12378-1 12573387

[CR25] Fairburn, C. G., Cooper, Z., & Shafran, R. (2003). Cognitive behaviour therapy for eating disorders: A transdiagnostic theory and treatment [Research Support, Non-U.S. Gov’t]. *Behaviour Research and Therapy*, *41*(5), 509–528. http://www.ncbi.nlm.nih.gov/pubmed/12711261 12711261 10.1016/s0005-7967(02)00088-8

[CR27] Fassino, S., Piero, A., Gramaglia, C., & Abbate-Daga, G. (2004). Clinical, psychopathological and personality correlates of interoceptive awareness in anorexia nervosa, bulimia nervosa and obesity. *Psychopathology*, *37*(4), 168–174. 10.1159/000079420 15237246 10.1159/000079420

[CR28] Fiene, L., & Brownlow, C. (2015). Investigating interoception and body awareness in adults with and without autism spectrum disorder. *Autism Research*, *8*(6), 709–716. 25808391 10.1002/aur.1486

[CR29] Forbush, K. T., Hagan, K. E., Kite, B. A., Chapa, D. A., Bohrer, B. K., & Gould, S. R. (2017). Understanding eating disorders within internalizing psychopathology: A novel transdiagnostic, hierarchical-dimensional model. *Compr Psychiatry*, *79*, 40–52. 28755757 10.1016/j.comppsych.2017.06.009

[CR30] Gabriele, E., Spooner, R., Brewer, R., & Murphy, J. (2021). Dissociations between self-reported interoceptive accuracy and attention: Evidence from the interoceptive attention scale. *Biological Psychology*, 108243. 10.1016/j.biopsycho.2021.108243 34929353

[CR31] Garfinkel, S. N., Tiley, C., O’Keeffe, S., Harrison, N. A., Seth, A. K., & Critchley, H. D. (2016). Discrepancies between dimensions of interoception in autism: Implications for emotion and anxiety. *Biological Psychology*, *114*, 117–126. 26724504 10.1016/j.biopsycho.2015.12.003

[CR32] Garner, D. M., Olmstead, M. P., & Polivy, J. (1983). Development and validation of a multidimensional eating disorder inventory for anorexia nervosa and bulimia. *International Journal of Eating Disorders*, *2*(2), 15–34.

[CR33] Ghasemi, A., & Zahediasl, S. (2012). Normality tests for statistical analysis: A guide for non-statisticians. *International Journal of Endocrinology and Metabolism*, *10*(2), 486. 23843808 10.5812/ijem.3505 PMC3693611

[CR34] Goldschmidt, J. (2018). A broad view: Disordered eating on the Autism Spectrum. *Eating Disorders Review*, *29*(3).

[CR35] Goldschmidt, J., & Song, H. J. (2015). At-risk and underserved: A proposed role for nutrition in the adult trajectory of autism. *J Acad Nutr Diet*, *115*(7), 1041–1047. 25840938 10.1016/j.jand.2015.02.013

[CR36] Grilo, C. M. (2013). Why no cognitive body image feature such as overvaluation of shape/weight in the binge eating disorder diagnosis? *International Journal of Eating Disorders*, *46*(3), 208–211. 23233198 10.1002/eat.22082 PMC3600067

[CR37] Grilo, C. M., Masheb, R. M., & White, M. A. (2010). Significance of overvaluation of shape/weight in binge-eating disorder: Comparative study with overweight and bulimia nervosa. *Obesity (Silver Spring)*, *18*(3), 499–504. 19713949 10.1038/oby.2009.280 PMC2845446

[CR38] Hair, J. F., Anderson, R. E., Babin, B. J., & Black, W. C. (2010). *Multivariate data analysis: A global perspective* (Vol. 7). In: Pearson.

[CR907] Harris, H. A., Derks, I. P., Prinzie, P., Louwerse, A., Hillegers, M. H., & Jansen, P. W. (2023). Interrelated development of autism spectrum disorder symptoms and eating problems in childhood: A population-based cohort. *Frontiers in Pediatrics, 11*, 1062012. 10.3389/fped.2023.1062012 PMC10185905 37205222

[CR39] Hatfield, T. R., Brown, R. F., Giummarra, M. J., & Lenggenhager, B. (2019). Autism spectrum disorder and interoception: Abnormalities in global integration? *Autism*, *23*(1), 212–222. 29139302 10.1177/1362361317738392

[CR41] Herbert, B. M., & Pollatos, O. (2012). The body in the mind: On the relationship between interoception and embodiment. *Topics in Cognitive Science*, *4*(4), 692–704. 22389201 10.1111/j.1756-8765.2012.01189.x

[CR40] Herbert, B. M., Blechert, J., Hautzinger, M., Matthias, E., & Herbert, C. (2013). Intuitive eating is associated with interoceptive sensitivity. Effects on body mass index. *Appetite*, *70*, 22–30. 23811348 10.1016/j.appet.2013.06.082

[CR42] Hobson, H., Westwood, H., Conway, J., McEwen, F. S., Colvert, E., Catmur, C., Bird, G., & Happé, F. (2020). Alexithymia and autism diagnostic assessments: Evidence from twins at genetic risk of autism and adults with anorexia nervosa. *Research in Autism Spectrum Disorders*, *73*, 101531.

[CR43] Hoiles, K. J., Egan, S. J., & Kane, R. T. (2012). The validity of the transdiagnostic cognitive behavioural model of eating disorders in predicting dietary restraint. *Eating Behaviors*, *13*(2), 123–126. 22365794 10.1016/j.eatbeh.2011.11.007

[CR5] http://www.ncbi.nlm.nih.gov/pubmed/11439754

[CR44] Hughes, E. K., Goldschmidt, A. B., Labuschagne, Z., Loeb, K. L., Sawyer, S. M., & Le Grange, D. (2013). Eating disorders with and without comorbid depression and anxiety: Similarities and differences in a clinical sample of children and adolescents. *European Eating Disorders Review: The Journal of the Eating Disorders Association*, *21*(5), 386–394. 10.1002/erv.2234 23681932 10.1002/erv.2234

[CR45] Jenkinson, P. M., Taylor, L., & Laws, K. R. (2018). Self-reported interoceptive deficits in eating disorders: A meta-analysis of studies using the eating disorder inventory. *Journal of Psychosomatic Research*. 10.1016/j.jpsychores.2018.04.005 29764604

[CR46] Kalyva, E. (2009). Comparison of eating attitudes between adolescent girls with and without Asperger Syndrome: Daughters’ and mothers’ reports. *Journal of Autism and Developmental Disorders*, *39*(3), 480–486. 10.1007/s10803-008-0648-5 18780173 10.1007/s10803-008-0648-5

[CR47] Karlsson, L., Rastam, M., & Wentz, E. (2013). The SWedish Eating Assessment for Autism spectrum disorders (SWEAA)-Validation of a self-report questionnaire targeting eating disturbances within the autism spectrum. *Research in Developmental Disabilities*, *34*(7), 2224–2233. 10.1016/j.ridd.2013.03.035 23643773 10.1016/j.ridd.2013.03.035

[CR49] Khalsa, S. S., & Lapidus, R. C. (2016). Can interoception improve the pragmatic search for biomarkers in psychiatry? *Frontiers in Psychiatry*, *7*, 121. 27504098 10.3389/fpsyt.2016.00121 PMC4958623

[CR50] Kinnaird, E., Norton, C., Pimblett, C., Stewart, C., & Tchanturia, K. (2019a). Eating as an autistic adult: An exploratory qualitative study. *PLoS One*, *14*(8). 10.1371/journal.pone.0221937 PMC6715205 31465510

[CR51] Kinnaird, E., Norton, C., Stewart, C., & Tchanturia, K. (2019b). Same behaviours, different reasons: What do patients with co-occurring anorexia and autism want from treatment? *International Review of Psychiatry*, 1–10. 10.1080/09540261.2018.1531831 30821179

[CR52] Kinnaird, E., Stewart, C., & Tchanturia, K. (2019c). Investigating alexithymia in autism: A systematic review and meta-analysis. *European Psychiatry*, *55*, 80–89. 30399531 10.1016/j.eurpsy.2018.09.004 PMC6331035

[CR53] Kinnaird, E., Stewart, C., & Tchanturia, K. (2020). Interoception in anorexia nervosa: Exploring associations with alexithymia and autistic traits. *Frontiers in Psychiatry*, *11*, 64. 32153442 10.3389/fpsyt.2020.00064 PMC7047099

[CR54] Koch, S. V., Larsen, J. T., Mouridsen, S. E., Bentz, M., Petersen, L., Bulik, C., Mortensen, P. B., & Plessen, K. J. (2015). Autism spectrum disorder in individuals with anorexia nervosa and in their first- and second-degree relatives: Danish nationwide register-based cohort-study. *British Journal of Psychiatry*. 10.1192/bjp.bp.114.153221 10.1192/bjp.bp.114.153221 25657359

[CR55] Lai, M. C., Lombardo, M. V., Auyeung, B., Chakrabarti, B., & Baron-Cohen, S. (2015). Sex/Gender differences and autism: Setting the scene for Future Research. *Journal of the American Academy of Child and Adolescent Psychiatry*, *54*(1), 11–24. 10.1016/j.jaac.2014.10.003 25524786 10.1016/j.jaac.2014.10.003 PMC4284309

[CR56] Lampard, A. M., Tasca, G. A., Balfour, L., & Bissada, H. (2013). An evaluation of the transdiagnostic cognitive-behavioural model of eating disorders. *European Eating Disorders Review*, *21*(2), 99–107. 23203942 10.1002/erv.2214

[CR58] Lord, C., Rutter, M., DiLavore, P. C., Risi, S., Gotham, K., & Bishop, S. I. (2012). *Autism Diagnostic Observation schedule, Second Edition (ADOS-2) manual (part 1) modules 1–4*. Western Psychological Services.

[CR57] Lord, C., Charman, T., Havdahl, A., Carbone, P., Anagnostou, E., Boyd, B., Carr, T., de Vries, P. J., Dissanayake, C., Divan, G., Freitag, C. M., Gotelli, M. M., Kasari, C., Knapp, M., Mundy, P., Plank, A., Scahill, L., Servili, C., Shattuck, P., & McCauley, J. B. (2022). The Lancet Commission on the future of care and clinical research in autism. *The Lancet*, *399*(10321), 271–334. 10.1016/s0140-6736(21)01541-5 10.1016/S0140-6736(21)01541-5 34883054

[CR59] Lovibond, S., & Lovibond, P. (1995). Depression Anxiety Stress Scale-21 (DASS-21). *Manual for the depression anxiety & stress scales*.

[CR60] Lucarelli, J., Pappas, D., Welchons, L., & Augustyn, M. (2017). Autism spectrum disorder and avoidant/restrictive food intake disorder. *Journal of Developmental & Behavioral Pediatrics*, *38*(1), 79–80. 27824638 10.1097/DBP.0000000000000362

[CR62] Mari-Bauset, S., Zazpe, I., Mari-Sanchis, A., Llopis-Gonzalez, A., & Morales-Suarez-Varela, M. (2014). Food selectivity in autism spectrum disorders: A systematic review [Review]. *Journal of Child Neurology*, *29*(11), 1554–1561. 10.1177/0883073813498821 24097852 10.1177/0883073813498821

[CR63] Marsero, S., Ruggiero, G. M., Scarone, S., Bertelli, S., & Sassaroli, S. (2011). The relationship between alexithymia and maladaptive perfectionism in eating disorders: A mediation moderation analysis methodology. *Eating and Weight Disorders-Studies on Anorexia Bulimia and Obesity*, *16*(3), E182–E187. 10.1007/BF03325130 22290034

[CR64] Merwin, R. M., Zucker, N. L., Lacy, J. L., & Elliott, C. A. (2010). Interoceptive awareness in eating disorders: Distinguishing lack of clarity from non-acceptance of internal experience. *Cognition and Emotion*, *24*(5), 892–902.

[CR103] Moseley, R., Shalev, I., Gregory, N., & Uzefovsky, F. (2024). Empathic disequilibrium as a predictor of nonsuicidal self-injury in autistic and nonautistic people. *Autism in Adulthood*. 10.1089/aut.2023.0134 PMC12851870 41625313

[CR65] Murphy, J., Brewer, R., Plans, D., Khalsa, S., Catmur, C., & Bird, G. (2018a). Validation of the interoceptive accuracy scale (IAS) supports distinction between self-reported interoceptive accuracy and awareness. *PsyArXiv Preprints*, *10*.

[CR66] Murphy, J., Catmur, C., & Bird, G. (2018b). Alexithymia is associated with a multidomain, multidimensional failure of interoception: Evidence from novel tests. *Journal of Experimental Psychology: General*, *147*(3), 398. 29154612 10.1037/xge0000366 PMC5824617

[CR67] Murphy, J., Catmur, C., & Bird, G. (2019). Classifying individual differences in interoception: Implications for the measurement of interoceptive awareness. *Psychonomic Bulletin & Review*, *26*(5), 1467–1471. 31270764 10.3758/s13423-019-01632-7 PMC6797703

[CR68] Nazar, B. P., Peynenburg, V., Rhind, C., Hibbs, R., Schmidt, U., Gowers, S., Macdonald, P., Goddard, E., Todd, G., & Micali, N. (2018). An examination of the clinical outcomes of adolescents and young adults with broad autism spectrum traits and autism spectrum disorder and anorexia nervosa: A multi centre study. *International Journal of Eating Disorders*. 10.1002/eat.22823 29331075

[CR69] Nemiah, J. C. (1976). Alexithymia: A view of the psychosomatic process. *Modern Trends in Psychosomatic Medicine*, *3*, 430–439.

[CR99] Nisticò, V., Ingrosso, G., Lombardi, F., Chiudinelli, E., Bianchini, G., Faggioli, R., Bertani, A., Gambini, O., & Demartini, B. (2024). Autistic traits, sensory sensitivity and eating disturbances in a sample of young adults referring to a generalized mental health clinic. *Eating and Weight Disorders-Studies on Anorexia, Bulimia and Obesity, 29*(1), 10. 10.1007/s40519-024-01639-7 PMC10806192 38261082

[CR70] Nowakowski, M. E., McFarlane, T., & Cassin, S. (2013). Alexithymia and eating disorders: A critical review of the literature. *J Eat Disord*, *1*, 21. 10.1186/2050-2974-1-21 24999402 10.1186/2050-2974-1-21 PMC4081716

[CR71] Oakley, B. F., Brewer, R., Bird, G., & Catmur, C. (2016). Theory of mind is not theory of emotion: A cautionary note on the reading the mind in the eyes test. *Journal of Abnormal Psychology*, *125*(6), 818. 27505409 10.1037/abn0000182 PMC4976760

[CR72] Oldershaw, A., Hambrook, D., Stahl, D., Tchanturia, K., Treasure, J., & Schmidt, U. (2011). The socio-emotional processing stream in Anorexia Nervosa. *Neuroscience and Biobehavioral Reviews*, *35*(3), 970–988. 10.1016/j.neubiorev.2010.11.001 21070808 10.1016/j.neubiorev.2010.11.001

[CR73] Oswald, A., Chapman, J., & Wilson, C. (2017). Do interoceptive awareness and interoceptive responsiveness mediate the relationship between body appreciation and intuitive eating in young women? *Appetite*, *109*, 66–72. 27866989 10.1016/j.appet.2016.11.019

[CR75] Phillips, K. L., Schieve, L. A., Visser, S., Boulet, S., Sharma, A. J., Kogan, M. D., Boyle, C. A., & Yeargin-Allsopp, M. (2014). Prevalence and impact of unhealthy weight in a national sample of US adolescents with autism and other learning and behavioral disabilities. *Maternal and Child Health Journal*, *18*(8), 1964–1975. 10.1007/s10995-014-1442-y 24553796 10.1007/s10995-014-1442-y PMC5328414

[CR76] Pooni, J., Ninteman, A., Bryant-Waugh, R., Nicholls, D., & Mandy, W. (2012). Investigating autism spectrum disorder and autistic traits in early onset eating disorder. *International Journal of Eating Disorders*, *45*(4), 583–591. 10.1002/eat.20980 22331792 10.1002/eat.20980

[CR77] Porges, S. (1993). Body perception questionnaire. *Laboratory of Developmental Assessment, University of Maryland*.

[CR78] Quattrocki, E., & Friston, K. (2014). Autism, oxytocin and interoception. *Neuroscience & Biobehavioral Reviews*, *47*, 410–430. 25277283 10.1016/j.neubiorev.2014.09.012 PMC4726659

[CR79] Råstam, M. (2008). Eating disturbances in autism spectrum disorders with focus on adolescent and adult years. *Clinical Neuropsychiatry*, *5*(1), 31–42. https://www.scopus.com/inward/record.uri?eid=2-s2.0-50249142402%26partnerID=40%26md5=47922db31a734e28cd524fe14add79ce

[CR80] Rhind, C., Bonfioli, E., Hibbs, R., Goddard, E., Macdonald, P., Gowers, S., Schmidt, U., Tchanturia, K., Micali, N., & Treasure, J. (2014). An examination of autism spectrum traits in adolescents with anorexia nervosa and their parents. *Mol Autism*, *5*(1), 56. 10.1186/2040-2392-5-56 25553237 10.1186/2040-2392-5-56 PMC4280745

[CR81] Schandry, R. (1981). Heart beat perception and emotional experience. *Psychophysiology*, *18*(4), 483–488. 7267933 10.1111/j.1469-8986.1981.tb02486.x

[CR82] Schauder, K. B., Mash, L. E., Bryant, L. K., & Cascio, C. J. (2015). Interoceptive ability and body awareness in autism spectrum disorder. *Journal of Experimental Child Psychology*, *131*, 193–200. 25498876 10.1016/j.jecp.2014.11.002 PMC4303499

[CR83] Sedgewick, F., Leppanen, J., & Tchanturia, K. (2020). Autistic adult outcomes on weight and body mass index: A large-scale online study. *Eating and Weight Disorders-Studies on Anorexia, Bulimia and Obesity, 25*, 795–801. 10.1007/s40519-019-00695-8 PMC7256089 31065975

[CR84] Spek, A. A., van Rijnsoever, W., van Laarhoven, L., & Kiep, M. (2019). Eating problems in men and women with an Autism Spectrum Disorder. *Journal of Autism and Developmental Disorders*, 1–8. 10.1007/s10803-019-03931-3 30798478

[CR85] Stewart, C. S., McEwen, F. S., Konstantellou, A., Eisler, I., & Simic, M. (2017). Impact of ASD traits on treatment outcomes of eating disorders in girls. *European Eating Disorders Review: The Journal of the Eating Disorders Association*, *25*(2), 123–128. 10.1002/erv.2497 28058799 10.1002/erv.2497

[CR87] Taylor, G. J., Bagby, R. M., & Parker, J. D. (1999). *Disorders of affect regulation: Alexithymia in medical and psychiatric illness*. Cambridge University Press.

[CR86] Taylor, E. C., Livingston, L. A., Clutterbuck, R. A., Shah, P., & Payne, C. (2020). Psychometric concerns with the 10-item Autism-Spectrum Quotient (AQ10) as a measure of trait autism in the general population. *Experimental Results*, *1*.

[CR88] Tchanturia, K., Adamson, J., Leppanen, J., & Westwood, H. (2017). Characteristics of autism spectrum disorder in anorexia nervosa: A naturalistic study in an inpatient treatment programme. *Autism*, 1362361317722431. 10.1177/1362361317722431 29105513

[CR89] Vagni, D., Moscone, D., Travaglione, S., & Cotugno, A. (2016). Using the Ritvo Autism Asperger Diagnostic Scale-revised (RAADS-R) disentangle the heterogeneity of autistic traits in an Italian eating disorder population. *Research in Autism Spectrum Disorders*, *32*, 143–155. 10.1016/j.rasd.2016.10.002

[CR90] Volpe, U., Tortorella, A., Manchia, M., Monteleone, A. M., Albert, U., & Monteleone, P. (2016). Eating disorders: What age at onset? *Psychiatry Research*, *238*, 225–227. 10.1016/j.psychres.2016.02.048 27086237 10.1016/j.psychres.2016.02.048

[CR91] Weston, R., & GoreJr, P. A. (2006). A brief guide to structural equation modeling. *The Counseling Psychologist*, *34*(5), 719–751.

[CR98] Westwood, H., & Tchanturia, K. (2017). Autism spectrum disorder in anorexia nervosa: An updated literature review. *Current Psychiatry Reports*, *19*(7), 41. 28540593 10.1007/s11920-017-0791-9 PMC5443871

[CR92] Westwood, H., Eisler, I., Mandy, W., Leppanen, J., Treasure, J., & Tchanturia, K. (2016a). Using the autism-spectrum quotient to measure autistic traits in Anorexia Nervosa: A systematic review and Meta-analysis. *Journal of Autism and Developmental Disorders*, *46*(3), 964–977. 10.1007/s10803-015-2641-0 26542816 10.1007/s10803-015-2641-0 PMC4746216

[CR94] Westwood, H., Lawrence, V., Fleming, C., & Tchanturia, K. (2016b). Exploration of Friendship experiences, before and after illness onset in females with Anorexia Nervosa: A qualitative study. *PLoS One*, *11*(9), e0163528. 10.1371/journal.pone.0163528 27676072 10.1371/journal.pone.0163528 PMC5038934

[CR97] Westwood, H., Stahl, D., Mandy, W., & Tchanturia, K. (2016c). The set-shifting profiles of anorexia nervosa and autism spectrum disorder using the Wisconsin Card sorting test: A systematic review and meta-analysis. *Psychological Medicine*, 1–19. 10.1017/S0033291716000581 10.1017/S0033291716000581 27109830

[CR93] Westwood, H., Kerr-Gaffney, J., Stahl, D., & Tchanturia, K. (2017a). Alexithymia in eating disorders: Systematic review and meta-analyses of studies using the Toronto Alexithymia Scale. *Journal of Psychosomatic Research*, *99*, 66–81. 28712432 10.1016/j.jpsychores.2017.06.007 PMC5986724

[CR95] Westwood, H., Mandy, W., Simic, M., & Tchanturia, K. (2017b). Assessing ASD in adolescent females with Anorexia Nervosa using clinical and developmental measures: A preliminary investigation. *Journal of Abnormal Child Psychology*. 10.1007/s10802-017-0301-x 10.1007/s10802-017-0301-x PMC5770498 28417276

[CR96] Westwood, H., Mandy, W., & Tchanuria, K. (2017c). Clinical evaluation of autistic symptoms in women with anorexia nervosa. *Mol Autism*. 10.1186/s13229-017-0128-x 28331571 10.1186/s13229-017-0128-x PMC5356303

