# Supplementary material for: The Relationship Between Interoception, Alexithymia, Autistic Traits and Eating Pathology in Autistic Adults
Source: J Autism Dev Disord. 2025 Feb 14;56(7):2574–87. doi: 10.1007/s10803-024-06708-5 (PMC13346133; doi:10.1007/s10803-024-06708-5)
Supplement: Supplementary file 1 — Supplementary Material 1 [file 10803_2024_6708_MOESM1_ESM.docx]

# Supporting information

**Demographic Information by Group.**

| Variable | autistic (*n*=191) | non-autistic (*n*=206) |
| --- | --- | --- |
| Sex assigned at birth  Male  Female | 47(24.61%)  144(75.39%) | 45(21.84%)  161(78.16%) |
| Gender Identify  Male  Female  Transgender  Non-conforming  Other (not listed) | 44(23.04%)  128(67.02%)  1(0.52%)  15(7.85%)  3(1.57%) | 46(22.33%)  156(75.73%)  0(0.00%)  2(0.97%)  2(0.97%) |
| Ethnicity  White  Asian  Black  Mixed  Other | 181(94.76%)  2(1.05%)  1(0.52%)  3(1.57%)  4(2.09%) | 156(75.73%)  25(12.14%)  5(2.43%)  12(5.83%)  8(3.88%) |
| Employment Status  Employed full-time  Employed part-time  Self-employed  Full-time education  Unemployed  Retired  Long-term sick | 48(25.13%)  24(12.57%)  14(7.33%)  26(13.61%)  29(15.18%)  10(5.24%)  40(20.94%) | 83(40.29%)  19(9.22%)  9(4.37%)  74(35.92%)  19(9.22)  2(0.97%)  0(0.00%) |
| Highest Level of Education  GCSE  A Level  Undergraduate Degree  Postgraduate degree  PhD/Doctorate  Not stated | 19(9.95%)  44(23.04%)  46(24.08%)  59(30.89%)  12(6.28%)  11(5.76%) | 6(2.91%)  100(48.54%)  44(21.36%)  43(20.87%)  11(5.34%)  2(0.97%) |
| Weight category  Underweight (BMI >18.5)  Healthy weight (BMI = 18.5-25)  Overweight (BMI = 25-30)  Obese (BMI > 30)  Not stated | 26(13.61%)  77(40.31%)  37(19.37%)  44(23.04%)  7(3.66%) | 16(7.77%)  133(64.56%)  34(16.50%)  17(8.25%)  6(2.91%) |
| Previous or current  ED Diagnoses  Females  Males | 48(25.13%)  46 (24.08%)  2 (1.05%) | 16(7.77%)  16 (7.77%)  0 (0%) |
| Previous or current psychiatric disorder  By category  Mood disorder  Anxiety disorder  Anxiety and depression  Psychotic disorder  Personality disorder  Multiple diagnoses  Not specified | 151(79.06%)  18(9.42%)  18(9.42%)  43(22.51%)  0(0.00%)  0(0.00%)  61(31.94%)  1(0.53%) | 64(31.07%)  12(5.83%)  15(7.28%)  9(4.37%)  0(0.00%)  1(0.49%)  22(10.68%)  1(0.49%) |

Data screening. For the entire sample there were 75 missing data values (i.e., individual values on one question on a questionnaire), representing < .01% of total data. For scales used within the SEM model, missing data values were imputed with the median of the group (autistic or non-autistic). Median imputation was used because the variables were ordinal. Missing data for continuous variables (weight and height) and categorical variables (level of education) were not imputed and excluded from analysis. As all variables included in the model were based on Likert scale responses, there was no reason to exclude them based on skewness unless they exhibited no variance. Thus, kurtosis was tested, with values greater than 1.00 or less than 1.00 indicating potentially problematic kurtosis (insufficient variance). AQ-10 total score had a borderline kurtosis issues (-1.41) but this was expected given that the AQ-10 is used to categorise respondents as displaying clinical levels of autistic traits, and thus no further action was taken.

Exploratory Factor Analysis. For the DASS21 and SWEAA, exploratory factor analysis (EFA) was conducted using Maximum Likelihood and Promax rotation to determine whether the subscale scores loaded together as expected, were adequately correlated and met criteria for validity and reliability, described in detail below. The pattern matrix for the final two factor model is displayed in Table 1. The Kaiser-Meyer-Olkin measure of sampling adequacy was .87, above the commonly recommended value of .6 (Field, 2009) and Bartlett’s Test of sphericity was significant, (approximate χ^2^(28) = 1801.94, *p* < .001). Communalities for all variables were sufficiently high (ranging from .36 to .82), thus indicating that the chosen subscale scores from the SWEAA were adequately correlated for a factor analysis. The reproduced matrix had 7% non-redundant residuals greater than .05, further confirming the adequacy of the variables and a 2-factor model.

The Cronbach’s alphas for the extracted factors were .83 and .89 for atypical eating and mood, respectively, indicating reflective specification (Jarvis et al., 2003). The factors demonstrated sufficient convergent validity as their loads were all above the recommended minimum threshold of .30 for a sample size of above 350 (Hair et al., 2010). The factors showed sufficient discriminant validity as the correlation between the two factors was < .07 (.064) and there were no cross-loadings. This two-factor model had a total variance explained of 62.37%, with all extracted factors having eigenvalues above 1.0.

**Table 1**

*Pattern Matrix for SWEAA and DASS subscales*

| Subscale | Factor 1 | Factor 2 |
| --- | --- | --- |
| SWEAA Eating Behaviour | .87 |  |
| SWEAA Mealtime Surroundings | .81 |  |
| SWEAA Social Situation at Mealtime | .69 |  |
| SWEAA Purchase of Food | .62 |  |
| SWEAA Simultaneous Capacity | .60 |  |
| DASS Depression |  | .91 |
| DASS Anxiety |  | .89 |
| DASS Stress |  | .78 |
| DASS = depression, anxiety stress scales; SWEAA = SWedish Eating Assessment for Autism spectrum disorders | | |

Confirmatory Factor Analysis. CFA led to the SWEAA Mealtime Surroundings subscale being removed due to poor fit. Modification indices were used to determine if the model could be improved. Accordingly, adjustment to the model was made by covarying the error terms of SWEAA Purchase Food and Eating Behaviour. Goodness of fit indices for the measurement model are displayed in Table 2, indicating that the measurement model was sufficient.

**Table 2**

*Goodness of Fit Metrics for CFA*

| Metric | Observed Value | Recommended* |
| --- | --- | --- |
| cmin/df | 2.09 | Between 1 & 3 |
| CFI | .99 | > .95 |
| RMSEA | .05 | < . 06 |
| PCLOSE | .40 | > .05 |
| SRMR | .02 | < .09 |
| *Byrne, 2016; CFI = Comparative Fix Index; cmin/df = minimum discrepancy divided by degrees of freedom; PCLOSE = a "p value" for testing the null hypothesis that the population RMSEA is no greater than .05; RMSEA = Root Mean Square Error of Approximation; SRMR = Standardized Root Mean Square Residual | | |

Convergent validity was tested by calculating the Average Variance Extracted (AVE), which was .75 for mood and .45 for atypical eating. While values should be > .50, Malhotra & Dash (2011) argue that AVE is often too strict, and reliability can be established through Composite Reliability (CR) alone. As the CR value was > .07 (.77), it was therefore deemed admissible. Discriminant validity was tested by comparing the square root of the AVE to the inter-factor correlation. As the square root values were ≥ the inter-factor correlation, discriminant validity was confirmed.

As data for both the dependent variable (EDE-Q global scores) and the independent variables were collected using the same survey, a common method bias test was performed to determine whether the method bias was affecting the results of the measurement model. The unmeasured latent factor method was used, recommended by Podsakoff et al. (2003) for studies that do not explicitly measure a common factor. Comparing the standardized regression weights before and after adding the Common Latent Factor (CLF) showed that none of the regression weights were significantly affected by the CLF, indicating no common method bias, therefore the CLF was not retained for the structural model.

As the plan was to moderate the structural model with a categorical grouping variable, configural and metric invariance tests were also conducted on the measurement model. The model of the unconstrained measurement model (with autistic and non-autistic groups loaded separately) had adequate fit (cmin/df = 1.64, CFI = .99, RMSEA = .04, SRMR = .04), indicating that the model was configurally invariant. After constraining the models to be equal, a chi-square difference test (Δχ^2^) was found to be non-significant (*p* = .086), thus the measurement model met criteria for metric invariance across groups.

Structural Model. SEM is typically used to analyse structural theory representing causal processes (Byrne, 2016), however, while SEM is *based* on causal hypotheses, it is not designed to *confirm* causal hypotheses and there is nothing inherent in SEM which prevents it being used with cross-sectional correlational data (Kelloway, 1995). Composite variables were created using factor scores in AMOS for atypical eating and mood. As all other variables in the model were manifest, the structural model consisted of a mixture of composite and manifest variables. The presence of influential outliers was examined using Cook’s distance analysis by running linear regressions between all independent variables and EDE-Q scores. It revealed no records exhibiting abnormal Cook’s distances (all < .08), indicating no influential outliers. Multicollinearity was tested using the Variable Inflation Factor (VIF) for all the exogenous (independent) variables simultaneously. All VIF values were less than 3.0, indicating that the exogenous variables were all distinct.

The full structural model was evaluated using the maximum likelihood method. The model initially demonstrated poor fit, so adjustments were needed to achieve adequate fit. As the effect of age on EDE-Q had no effect different from 0 (*β* = -.001, SE = .01, CR = -.26), a direct path from age to EDE-Q to age was deleted to free up a parameter and gain a degree of freedom within the model. Examination of modification indices revealed that creating direct paths between mood and atypical eating, and between BPQ, TAS and EDE-Q would improve the model fit. As these relationships make theoretical sense, these steps were taken, which improved the model fit. Goodness of Fit metrics for structural model are displayed in Table 3.

**Table 3**

*Goodness of Fit Metrics for Structural Model*

| Metric | Observed Value | Recommended* |
| --- | --- | --- |
| cmin/df | 1.94 | Between 1 & 3 |
| CFI | .99 | > .95 |
| RMSEA | .05 | < . 06 |
| PCLOSE | .43 | > .05 |
| SRMR | .01 | < .09 |
| *Byrne, 2016; CFI = Comparative Fix Index; cmin/df = minimum discrepancy divided by degrees of freedom; PCLOSE = a "p value" for testing the null hypothesis that the population RMSEA is no greater than .05; RMSEA = Root Mean Square Error of Approximation; SRMR = Standardized Root Mean Square Residual | | |

As adjustments to the model led to the possibility of there being both direct and indirect effects of TAS-20 and BPQ on EDE-Q global scores due to the inclusion of direct paths from TAS-20 and BPQ to EDE-Q , the direct effects were analysed. The direct effect of TAS-20 scores on EDE-Q global scores was not significant (*β* = .033, 95% CI [-.08, .15], *p* = .670) but there was a significant direct effect of BPQ scores on EDE-Q scores (*β* = .160, 95% CI [.07, .25], *p* = .001). Therefore, when considering the mediation effect of atypical eating on the relationship between BPQ and EDE-Q global scores, both the indirect and direct effects were analysed for establishing potential partial mediation.


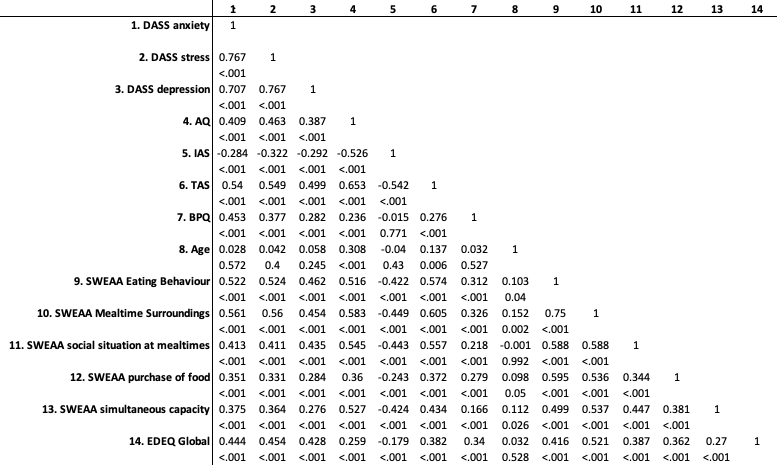
**Bivariate Correlations.** Simple correlations between the variables included in the SEM, with their associated *p* values below, are presented in the table below.

**Group differences in indirect effects**

This was conducted by creating nested models using the user-defined AMOS estimand, “MyIndirectEffect” (Gaskin, 2016). The test used 2000 bias-corrected bootstrapping resamples with 95% confidence intervals. This method multiplies the standardised regression coefficients of the indirect effect (e.g., AQ-10 ---> Atypical Eating and Atypical Eating ---> EDE-Q), providing a bias-corrected estimate with upper and lower bounds and a *p* value for the indirect effect. This analysis was conducted to test the indirect effect of atypical eating on AQ-10, IAS, TAS and BPQ scores with Bonferroni adjusted *α* of .0125 to account for multiple comparisons. Indirect effects are significant if their 95% CI does not include zero.

If the indirect effect for atypical eating on EDE-Q global scores was significant at a global level, then moderated mediation was conducted using the user defined AMOS estimand, “MyModMed” (Gaskin, 2016). This method tests whether the difference between indirect effects at each level of the moderator (autistic or non-autistic) is significantly different from zero, by subtracting the indirect effect of the autistic group from that of the non-autistic group. Again, variances were constrained to equality across the two groups and bootstrapping with 2000 bias-corrected resamples was applied.

#### Alternative Models. To test the robustness of the mediated model and in line with SEM recommendations (e.g., Weston & Gore, 2006), the structural model was compared with two alternative models that might theoretically explain the relationship between the variables. The first model was based on the theory that alexithymia may mediate the relationship between interoception and eating disturbances and thus, the TAS-20 was positioned as a mediator between the IAS/BPQ, and both atypical eating and EDE-Q global scores. The second competing model was based on the finding that interoceptive attention mediated the relationship between body appreciation and intuitive eating (Oswald et al., 2017) and therefore atypical eating was treated as an independent variable and both the BPQ and IAS were imputed as mediators in the relationship between atypical eating and EDE-Q scores. The original proposed structural model provided the best Goodness of Fit indices and so was preferred over the alternative models.

**Figure 1**


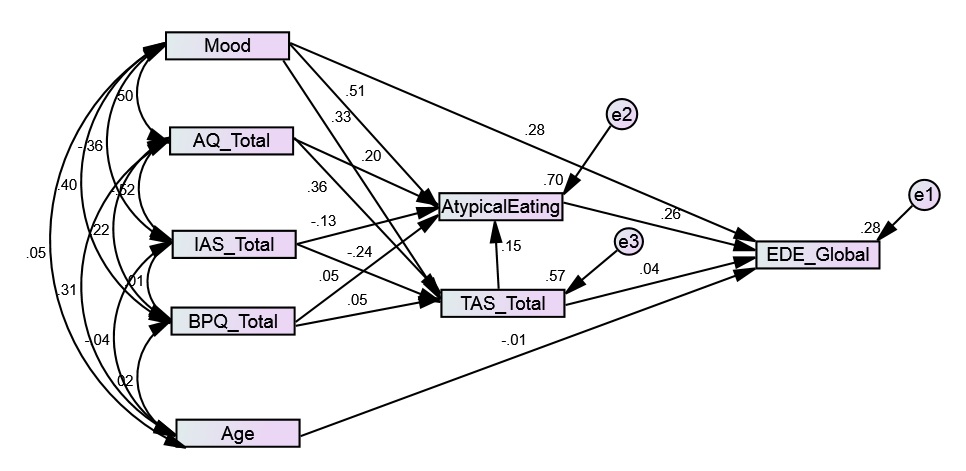
*Alternative Model 1*

**Table 4**

*Goodness of Fit Estimates for Alternative Model 1*

| Metric | Observed Value | Recommended* |
| --- | --- | --- |
| cmin/df | 3.77 | Between 1 & 3 |
| CFI | .99 | > .95 |
| RMSEA | .08 | < . 06 |
| PCLOSE | .07 | > .05 |
| SRMR | .03 | < .09 |
| *Byrne, 2016; CFI = Comparative Fix Index; cmin/df = minimum discrepancy divided by degrees of freedom; PCLOSE = a "p value" for testing the null hypothesis that the population RMSEA is no greater than .05; RMSEA = Root Mean Square Error of Approximation; SRMR = Standardized Root Mean Square Residual | | |

**Figure 2**


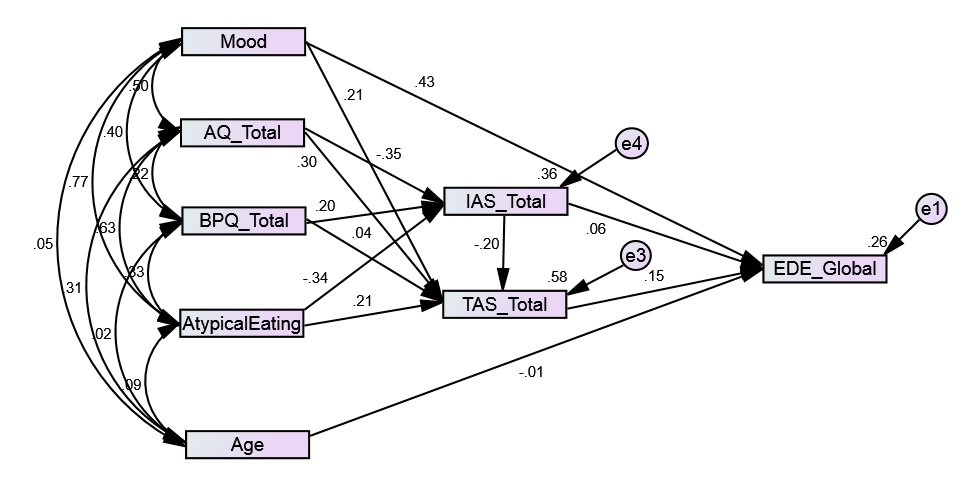
*Alternative Model 2*

**Table 5**

*Goodness of Fit Estimates for Alternative Model 2*

| Metric | Observed Value | Recommended* |
| --- | --- | --- |
| cmin/df | 5.90 | Between 1 & 3 |
| CFI | .98 | > .95 |
| RMSEA | .11 | < . 06 |
| PCLOSE | .002 | > .05 |
| SRMR | .03 | < .09 |
| *Byrne, 2016; CFI = Comparative Fix Index; cmin/df = minimum discrepancy divided by degrees of freedom; PCLOSE = a ‘p value’ for testing the null hypothesis that the population RMSEA is no greater than .05; RMSEA = Root Mean Square Error of Approximation; SRMR = Standardized Root Mean Square Residual | | |
